# Clinical-imaging and therapeutic evaluation of nodule-mass pulmonary cryptococcosis and pneumonia-type pulmonary cryptococcosis

**DOI:** 10.1186/s12890-025-03781-z

**Published:** 2025-07-02

**Authors:** Yanli Zhang, Ling Liu, Wei Li, Chao Ran

**Affiliations:** 1 Department of Clinical Pharmacy, Affiliated Hospital of Yangzhou University, Yangzhou University, Yangzhou, China; 2 Obstetrics Department, Affiliated Yantai Yuhuangding Hospital of Qingdao University, Yantai, China; 3 Medical Imaging Department, Affiliated Hospital of Yangzhou University, Yangzhou University, No. 368, Hanjiang Middle Road, Hanjiang District, Yangzhou, 225100 China; 4 Department of Radiology, Affiliated Yantai Yuhuangding Hospital of Qingdao University, Yantai, China

**Keywords:** Pulmonary cryptococcosis, Nodule-mass, Pneumonia-type, Computed tomography

## Abstract

**Background:**

The different morphological changes of pulmonary cryptococcosis (PC) cause difficulties in diagnosis and treatment. To evaluate the clinical-imaging and therapeutic differences between nodule-mass PC and pneumonia-type PC.

**Materials and methods:**

The clinical-imaging data of 68 patients with PC were collected, including nodule-mass PC (36 cases) and pneumonia-type PC (32 cases). Their clinical-imaging findings were retrospectively analyzed to determine the independent discriminators. Their diagnostic and therapeutic effects were compared.

**Results:**

Compared with nodule-mass PC, pneumonia-type PC was more common with respiratory symptoms (19.4% vs. 81.3%, *P* < 0.001), inflammatory response (16.7% vs. 71.9%, *P* < 0.001), bilateral distribution (16.7% vs. 68.8%, *P* < 0.001), mediastinal lymphadenopathy (0 vs. 21.9%, *P* = 0.010) and pleural effusion (0 vs. 25%, *P* = 0.005). While CT malignant signs were more common in nodule-mass PC (66.7% vs. 12.5%, *P* < 0.001). Multivariate logistic regression analysis showed respiratory symptoms, inflammatory response, bilateral distribution, and malignant CT signs were independent discriminators, with moderate areas under the curve (AUC, 0.760–0.809). Their combined efficacy was significantly improved, with the highest AUC (0.937). Unlike pneumonia-type PC, nodule-mass PC was often treated with low-dose, short-term monofluconazole treatment. However, both groups showed good therapeutic effects.

**Conclusion:**

Respiratory symptoms, inflammatory response, bilateral distribution, and malignant CT signs were different between nodule-mass PC and pneumonia-type pulmonary PC. Combining these independent discriminators could reveal its clinical-imaging diversity. The graded antifungal therapy based on CT morphology was effective.

## Introduction

Pulmonary cryptococcosis (PC) is an invasive fungal disease caused by cryptococcus, which can occur in acute, subacute, and chronic forms [1, 2]. With the application of glucocorticoids, antineoplastic drugs, and broad-spectrum antibiotics, the incidence of immunocompromised PC has increased [1]. Meanwhile, with the popularization of health examinations, puncture biopsy, and antigen detection, PC is also common in immunocompetent individuals [2]. The different immune states and inflammatory processes can complicate its clinical-imaging findings [3]. Previous studies mainly focused on the differentiation of PC from lung tumors or pneumonia, as well as the impact of different immune states on it [3–6]. Immune status has been an important reference for the diagnosis and treatment of PC, but its evaluation criteria are inconclusive. Nodule-mass is the most common morphological type with typical granulomatous inflammation during the stable period [4]. However, it is not unique, and pneumonia-type changes are not rare, especially in the acute phase [5, 6]. The morphological changes of PC cause difficulties in clinical practice. Due to its low incidence, a comprehensive clinical-imaging evaluation of pneumonia-type PC has not been performed. Therefore, this study aims to reveal the clinical-imaging diversity of PC through a comparison between nodule-mass PC and pneumonia-type PC. Independent discriminative factors are screened and validated to improve the clinical diagnostic level. Furthermore, the influence of different CT morphologies on treatment is evaluated.

## Materials and methods

### Clinical data

This study was approved by the Ethics Committee of our institutions. From June 2014 to December 2023, 68 patients with PC were collected. The inclusion criteria included definite pathological diagnosis, positive cryptococcal antigen (CrAg, colloidal gold labeled), or positive etiological culture. The exclusion criteria included incomplete clinical and imaging data, with other primary lesions, or infection with other pathogens. The immune status was comprehensively evaluated from three aspects: clinical indicators, humoral immune indicators, and cellular immune indicators [1, 7]. According to previous studies and our laboratory testing standards, the cut-off points of neutropenia, lymphopenia, humoral immune indicators, and cellular immune indicators were confirmed [1, 7]. Patients with one of the following conditions were considered as immunocompromised individuals. Clinical indicators: use of immunosuppressants or glucocorticoids (systemic glucocorticoids therapy, 5–40 mg/d based on prednisone, excluding inhaled glucocorticoids therapy), malignancies during radiochemotherapy, HIV infection, immunosuppressive diseases, organ transplantation, chronic wasting diseases, neutropenia (peripheral absolute neutrophil count < 2.0 × 109 cells/L) or lymphopenia (absolute lymphocyte count < 1,000 cells/µL). Humoral immune indicators: serum immunoglobulin IgG < 7.0 g/L, IgA < 0.4 g/L, or IgM < 0.7 g/L. Cellular immune indicators: the percentage of CD3 cells < 60%, the percentage of CD4 cells < 24.5%, the percentage of CD8 cells < 18.5%, or CD4/CD8 ratio < 1.02. Immunocompetent individuals did not have these potential risk factors. According to the CT findings at the first examination, these patients were divided into a nodule-mass PC group (36 cases) and a pneumonia-type PC group (32 cases). The around opacity with a maximum diameter ≤ 3 was defined as nodule PC. The around opacity with a maximum diameter >3 cm was defined as mass PC. Both of them constituted the nodule-mass PC. Pneumonia-type PC referred to patchy consolidation or interstitial opacity obscuring the underlying structures to varying degrees. The inflammatory response included one or more of the following indicators: white blood cell count > 10 × 10^9^/L, neutrophil count > 7 × 10^9^/L, neutrophil percentage > 70%, and C-reactive protein > 8.2 mg/L [1, 7].

### Imaging evaluations

Before treatment, GE Lightspeed 16 and GE Optima CT660 (GE Healthcare, Milwaukee, USA) were used in all patients for unenhanced scanning. The CT scanning parameters were as follows: tube voltage, 120 kV; tube current, 10–300 mA; spiral pitch, 1.0; collimation width, 16 × 0.625 mm or 64 × 0.625 mm. The built-in software was used for lung-window reconstruction (lung algorithm, reconstruction kernel = 60–80) and mediastinal-window reconstruction (standard algorithm, reconstruction kernel = 20–30) with a slice thickness of 1 mm and slice interval of 1 mm. Imaging evaluation was performed on the Medcare AnyImage workstation (V4.5). The PC lesions were analyzed on lung-window images (window width, 1000–2000 Hu; window level, -800 - -450 Hu). The pleural effusion and mediastinal lymphadenopathy were observed on mediastinal-window images (window width, 250–500 Hu; window level, 30–50 Hu). Imaging indicators included the distribution of lesions (unilateral or bilateral distribution), CT signs (lobulation, spiculation, vacuole, cavitation, pleural indentation, air-bronchogram and halo sign), pleural abnormalities (pleural involvement or pleural effusion) and mediastinal lymphadenopathy. The ground-glass opacity around the lesion was defined as a halo sign. Malignant CT signs included lobulation, spiculation, vacuole, cavitation, or pleural indentation. Pleural involvement referred to the focal or extensive pleural attachment of the lesion. A lymph node with a short axis diameter ≥ 10 mm was defined as lymphadenopathy. Imaging data were evaluated by two experienced thoracic radiologists with fifteen years of professional experience, without knowing the diagnoses. The radiologists interpreted the CT images with 1 mm slice thickness and 1 mm slice interval for detailed information. In case of disagreement, consensus should be reached through consultation.

### Therapeutic plan and evaluation

The treatment goal of PC is to control infection and prevent dissemination. According to the immune status, clinical symptoms, or cryptococcal dissemination, antifungal therapy was conducted [8–10]. Monofluconazole treatment (200–400 mg/d, 6–12 months) was recommended for asymptomatic and mild-moderate PC. Combined treatment was suggested for patients with severe symptoms, cryptococcal dissemination and immunodeficiency, including induction therapy (amphotericin B, 0.5-1.0 mg/kg/day and 5-fluorocytosine, 100 mg/kg/day, at least 4 weeks), consolidation therapy (fluconazole, 400 mg/day, 8 weeks), and maintenance therapy (fluconazole, 200 mg/day, 6–12 months).

Oral administration of fluconazole alone was a monofluconazole treatment. Fluconazole dosage less than 400 mg/d was defined as low-dose. Antifungal treatment lasting less than 6 months was considered short-term treatment [8–10]. The reappearance of the lesion after initial absorption was called relapse. The outcomes of PC were evaluated by clinical-imaging findings and therapeutic responses [8–10]. A good therapeutic effect was defined as the clinical-imaging improvement without relapse and persistent positive CrAg within the standard antifungal course. A poor therapeutic effect was defined as clinical-imaging deterioration, relapse, and cryptococcal dissemination within the standard antifungal course or death associated with cryptococcal infection.

### Statistical analysis

By IBM SPSS version 26.0 (SPSS, Chicago, IL), descriptive statistics were used to characterize the clinical-imaging features of both groups. All data were presented as numbers (n) or mean ± SD. An independent t-test (two-tailed) or Mann-Whitney U test was performed for the comparison of continuous variables between the two groups. The Chi-square test or Fisher’s exact test (two-tailed) was performed to assess the difference between dichotomous variables. For the inter-observer variability of CT analyses, the inter-class correlation coefficient (ICC) > 0.75 or Kappa ≥ 0.8 was considered a better agreement. Based on statistical significance and sample size, baseline analysis and multivariate logistic regression were performed to determine the independent differential factors between nodule-mass PC and pneumonia-type PC. For diagnostic value assessment, receiver operating characteristic (ROC) curve analysis and the Delong test were used to calculate and compare their areas under curves (AUC). *P* < 0.05 was defined as statistically significant.

## Results

### Clinical findings

In this study, respiratory symptoms included cough, expectoration, chest tightness, chest pain, fever, etc. Compared with nodule-mass PC, pneumonia-type PC was more common with respiratory symptoms (19.4% vs. 81.3%, *P* < 0.001) and inflammatory response (16.7% vs. 71.9%, *P* < 0.001). There was no difference in age, sex, environmental exposure, and immune state between the two groups. See Table 1 for details.

**Table 1:** Clinical-imaging comparison between nodule-mass PC and pneumonia-type PC

	Nodule-mass PC (*n* = 36)	Pneumonia-type PC (*n* = 32)	*P*
Sex (male/female)	22/14	18/14	0.684
Age (years)	47.6 ± 11.6	49.8 ± 12.8	0.329
Environmental exposure	5 (13.9%)	6 (18.8%)	0.587
Immunocompromised state	9 (25%)	11 (34.4%)	0.397
Respiratory symptom	7 (19.4%)	26 (81.3%)	< 0.001^*^
Inflammatory response	6 (16.7%)	23 (71.9%)	< 0.001^*^
Bilateral distribution	6 (16.7%)	22 (68.8%)	< 0.001^*^
Air-bronchogram	11 (30.6%)	13 (40.6%)	0.386
Halo sign	15 (41.7%)	10 (31.3%)	0.374
Malignant CT signs	24 (66.7%)	4 (12.5%)	< 0.001^*^
Pleural involvement	26 (72.2%)	20 (62.5%)	0.392
Pleural effusions	0	8 (25%)	0.005^*^
Mediastinal lymphadenopathy	0	7 (21.9%)	0.010^*^

PC, pulmonary cryptococcosis; CT, computed tomography. Values are given as n, n (%) or mean ± SD. *Significance values

Among the 68 patients in this study, 20 cases (29.4%) were immunocompromised PC, including 9 cases of nodule-mass PC and 11 cases of pneumonia-type PC. Most nodule-mass immunocompromised PC (7 cases, 77.8%) presented with mild-moderate nonspecific clinical symptoms, including cough, expectoration, chest tightness, and fever. All 11 cases of pneumonia-type immunocompromised PC had severe clinical symptoms, including high fever, dyspnea, hypoxemia, and acute respiratory failure. Most of the 48 immunocompetent PC patients (27 nodule-mass PC and 21 pneumonia-type PC) were asymptomatic clinical coincidences (33 cases, 68.8%), including nodule-mass PC 27 cases and pneumonia-type PC 6 cases.

### CT findings

**Fig. 1:**
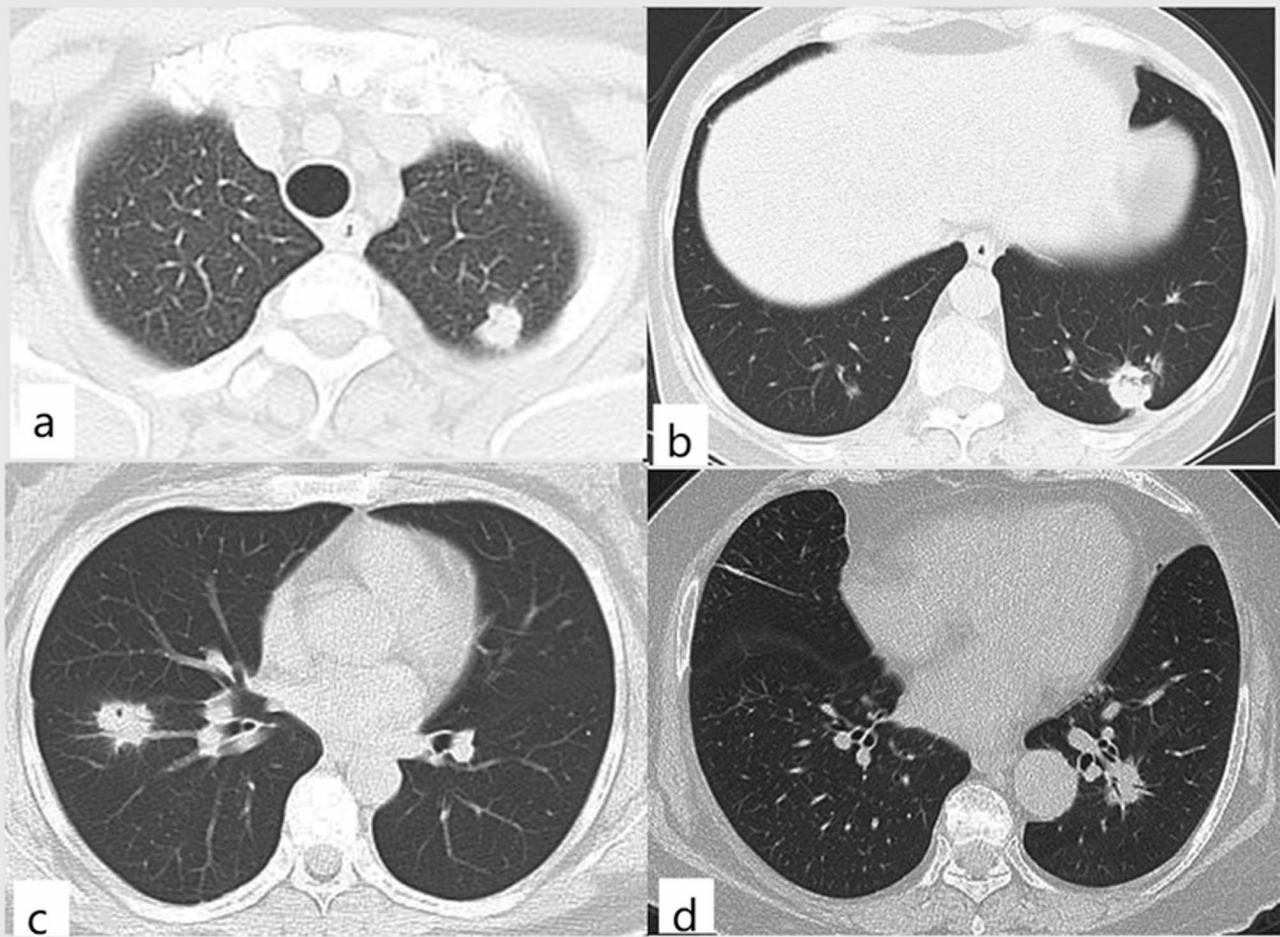
CT findings of nodule-mass PC. Axial CT image showed a lobulated nodule in the subpleural region of the left upper lobe (**a**). Axial CT image showed a nodule with vacuole, spiculation, and pleural indentation in the left lower lobe (**b**). Axial CT image showed a nodule with vacuole and spiculation in the right lower lobe (**c**). A nodule with spiculation and air-bronchogram could be seen in the left lower lobe on axial CT (**d**)

**Fig. 2:**
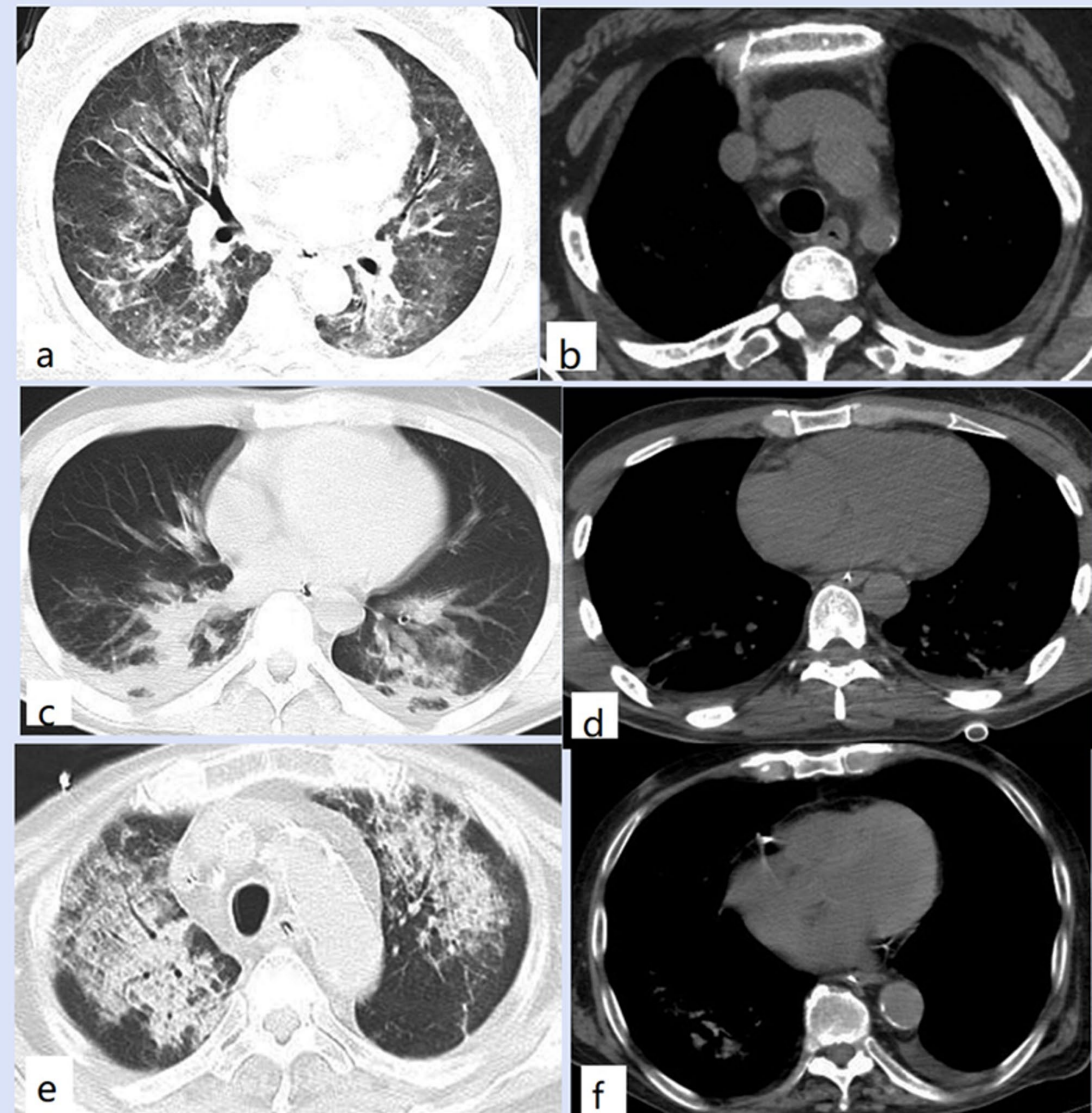
CT findings of pneumonia-type PC. Axial CT images showed bilateral ground-glass opacities with air-bronchogram (**a**), left pleural effusion, and mediastinal lymphadenopathy (**b**). Patchy consolidations could be seen in the bilateral lower lobes (**c**) and with bilateral pleural involvement and pleural effusions (**d**) on axial CT. Axial CT images showed diffuse consolidation-interstitial opacities in the bilateral upper lobes (**e**) with air-bronchogram (**e**) and left pleural effusion (**f**)

For these CT analyses, the inter-observer agreements between the two observers were good (ICC = 0.8373, Kappa = 0.8621). There were no differences in air-bronchograms (*P* = 0.386) and halo signs (*P* = 0.374) between the two groups. Malignant CT signs (lobulation, spiculation, vacuole, cavitation, or pleural indentation) were more common in nodule-mass PC (66.7% vs. 12.5%, *P* < 0.001). Pneumonia-type PC was more common with bilateral distribution (16.7% vs. 68.8%, *P* < 0.001) and mediastinal lymphadenopathy (0 vs. 21.9%, *P* = 0.010). Although the two groups showed similar pleural involvement (*P* = 0.392), pneumonia-type PC was more likely to cause pleural effusion (0 vs. 25%, *P* = 0.005). See Table 1; Figs. 1 and 2 for details.

### Selection and evaluation of independent discriminative factors

**Fig. 3:**
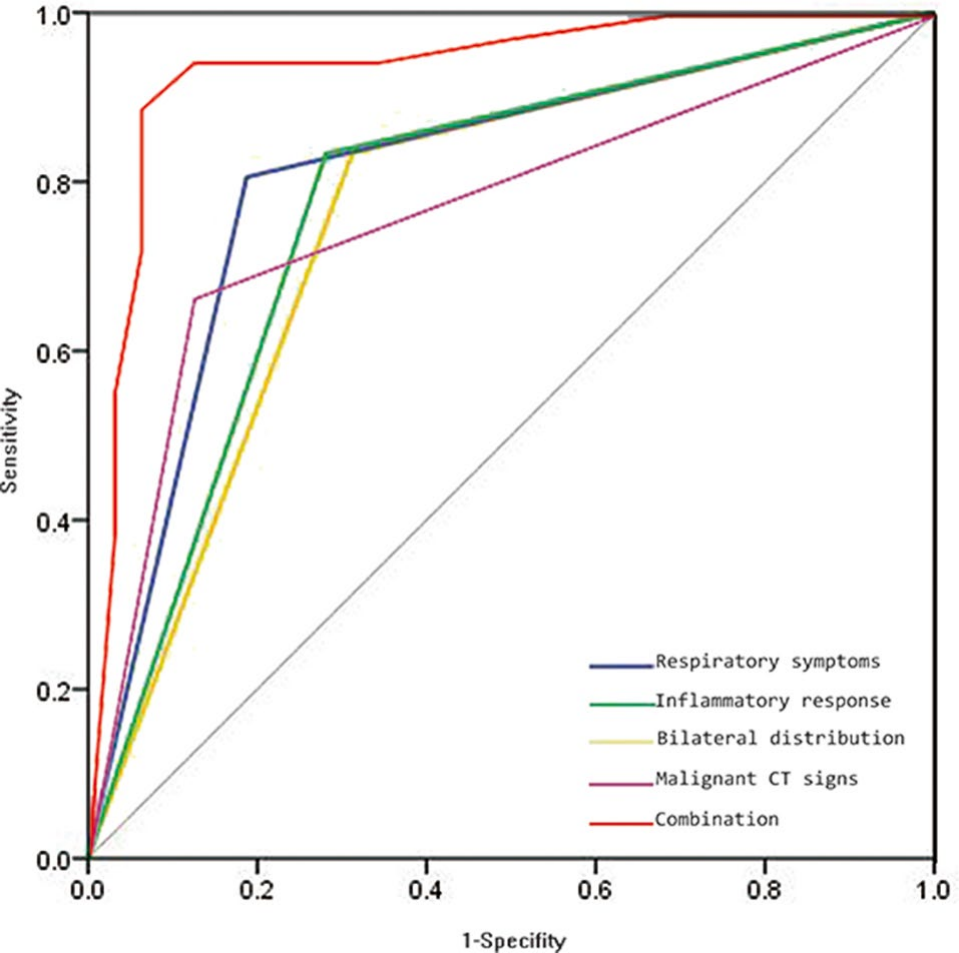
ROC curves of independent clinical-imaging features for PC differentiation. The combination parameter achieved the best diagnostic performance with an AUC value of 0.937

Multivariate logistic regression analysis showed respiratory symptoms, inflammatory response, bilateral distribution, and malignant CT signs were independent discriminators, with moderate AUC values (0.760–0.809). The Delong test showed that there was no significant difference in the discriminative power between these clinical-imaging features (*P* > 0.05). Combining respiratory symptoms, inflammatory responses, bilateral distribution, and malignant CT signs significantly improved differential performance, with a higher AUC value (0.937) than any single clinical-imaging feature (Delong test, *P* ≤ 0.002). See Tables 2 and 3; Fig. 3 for details.

**Table 2:** Clinical-imaging multivariate logistic regression analysis

	OR	95% CI for OR	*P*
Respiratory symptom	0.110	0.021–0.587	0.010^*^
Inflammatory response	0.133	0.025–0.709	0.018^*^
Malignant CT signs	6.755	1.110−41.085	0.038^*^
Bilateral distribution	0.148	0.028–0.794	0.026^*^

OR, Odds ratio; 95% CI, 95% confidence interval. *Significance values

**Table 3:** Diagnostic value of clinical-imaging parameters for both groups

	AUC	95% CI for AUC	*P*	AUC vs. combination
Respiratory symptom	0.809	0.700−0.918	< 0.001^*^	*P* = 0.002
Inflammatory response	0.776	0.660–0.892	< 0.001^*^	*P* = 0.001
Malignant CT signs	0.771	0.656–0.886	< 0.001^*^	*P* < 0.001
Bilateral distribution	0.760	0.641–0.879	< 0.001^*^	*P* = 0.001
Combination	0.937	0.873–0.999	< 0.001^*^	-

AUC, area under curve; 95% CI, 95% confidence interval. *Significance values

### Therapeutic effects

Most of the PC patients in this study (61/68, 89.7%) had good therapeutic effects. Two cases of nodule-mass PC and five cases of pneumonia-type PC had poor therapeutic effects, all of which were immunocompromised PC. Two cases of nodule-mass immunocompromised PC showed worsening clinical symptoms and enlarged lesions during treatment but improved after long-term high-dose combined treatment. Although five cases of immunocompromised pneumonia-type PC were treated with combined treatment, there were still 2 cases of recurrence, 2 cases of central nervous system dissemination, and 1 case of death.

Compared with pneumonia-type PC, nodule-mass PC was more common with short-term (88.9% vs. 9.4%) low-dose (86.1% vs. 12.5%) monofluconazole treatment (91.7% vs. 15.6%). However, both groups showed good therapeutic effects (94.4% vs. 84.4%) with few relapses (0 vs. 6.3%). See Table 4 for details.

**Table 4:** Therapeutic comparison between nodule-mass PC and pneumonia-type PC

	Nodule-mass PC (*n* = 36)	Pneumonia-type PC (*n* = 32)	*P*
Monofluconazole treatment	33 (91.7%)	5 (15.6%)	< 0.001^*^
Short-term treatment	32 (88.9%)	3 (9.4%)	< 0.001^*^
Low-dose treatment	31 (86.1%)	4 (12.5%)	< 0.001^*^
Good therapeutic effect	34 (94.4%)	27 (84.4%)	0.335
Relapse	0	2 (6.3%)	0.218

PC, pulmonary cryptococcosis. Values are given as n (%). *Significance values

## Discussion

Although PC was considered to have a male tendency [11], this study did not find any difference in sex and environmental exposure between the two groups. Clinically, PC is increasingly occurring in male and female patients without clear environmental exposure. The immune mechanism is complex in the pathogenesis of PC [12]. The ranges and degrees of PC can be influenced by immune status [5]. Immunocompromised patients cannot localize the lesion and develop cryptococcal pneumonia [13, 14]. However, the immune status is difficult to evaluate without unified criteria for immune function. Both groups of PC patients were more common in immunocompetent states. Maybe, pneumonia-type PC was in the acute infection stage, causing significant respiratory symptoms, inflammatory responses, and bilateral distribution [15, 16]. On the contrary, nodule-mass PC is a non-acute granuloma formation without clinical symptoms [15, 17].

Both nodule-mass PC and pneumonia-type PC showed the halo signs and air-bronchogram, but their formative mechanisms might be different. For nodule-mass PC, the halo sign was the localized inflammatory encapsulation of the lesion, while the air-bronchogram might be the bronchial ventilation during PC absorption [18, 19]. For pneumonia-type PC, obvious inflammatory exudation or cryptococcal aggregation could fill the alveoli and also form the halo sign and air-bronchogram [20]. Because of different inflammatory evolution, nodule-mass PC could develop some malignant CT signs (lobulation, spiculation, vacuole, cavitation, or pleural indentation) [6, 21]. The imaging follow-up is necessary to avoid misdiagnosis. Additionally, the air-bronchogram within the nodule-mass PC was different from neoplastic lesions, suggesting the bronchial structural integrity. Due to the similar airway inhalation and suitable growth conditions, Cryptococcus can colonize in the subpleural region [17, 22]. Although both PC groups showed pleural involvement, pleural effusion was more common in pneumonia-type PC. The obvious inflammatory response and cryptococcal invasiveness in the acute stage can damage the pleural-airway integrity and form pleural effusion [23]. Similarly, pneumonia-type PC involving the interstitial lymphatic vessels increased the possibility of mediastinal lymphadenopathy [24, 25].

Excluding confounding interactions, multivariate logistic regression analysis provided a reliable clinical-imaging interpretation for PC. Significant respiratory symptoms and inflammatory response represented the severity of pneumonia-type PC, and the bilateral distribution reflected its extensive involvement. Previously, these clinical-imaging features were believed to be related to immune deficiency [26, 27]. However, they might also be the potent inflammatory defense of immunocompetent individuals during the acute phase. Malignant CT signs were local findings of nodule-mass PC during absorption. Nodule-mass PC and pneumonia-type PC may be different stages of cryptococcal infection. Although these independent differential features showed moderate diagnostic performances, their combined application had the highest AUC value. Compared with the single indicator, the combined efficacy reflected the comprehensive consideration of clinical-imaging information. In addition, the clinical-imaging findings of PC are complex, and there may be high-order nonlinear relationships among these predictive predictors.

Previously, it was believed that pneumonia-type PC was more common in immunocompromised individuals, with severe symptoms and poor prognosis [14, 18, 20]. However, we found that pneumonia-type PC was not always associated with immune deficiency. Although most of the patients in this study were immunocompetent, the treatment plans for nodule-mass PC and pneumonia-type PC were different. Nodule-mass PC is in the chronic stable stage, macrophages engulf cryptococcus and its products, requiring only low-dose short-term monofluconazole treatment [9–11]. While the pneumonia-type is acute PC, with strong inflammatory reactions and severe clinical-imaging findings. Therefore, long-term high-dose combination therapy is needed [9–11]. Stratified treatment based on CT morphology is crucial and effective.

However, the treatment of immunocompromised PC remained challenging. Immunocompromised PC had more severe clinical manifestations and poorer therapeutic outcomes, especially for pneumonia-type PC. Its widespread distribution and immunodeficiency exacerbated acute respiratory failure and extrapulmonary dissemination, leading to long-term high-dose combined treatment [9–11]. Therefore, early diagnosis and treatment of PC is very important. Metagenomic next-generation sequencing (mNGS) and CrAg are advanced diagnostic techniques for cryptococcosis, providing useful decision-making information for clinicians. Compared to mNGS, CrAg has better economy, practicality, and specificity [28, 29]. Therefore, CrAg was regarded as one of the diagnostic criteria for PC in this study. In cases of immunodeficiency and ineffective antibacterial treatment, CrAg should be performed to clarify the diagnosis and provide reasonable treatment. During the antifungal process, CrAg could also evaluate the therapeutic effects and adjust the medication strategies.

This study was only focused on the morphological subtypes of confirmed PC. Community-acquired pneumonia (CAP) and pneumonia-type PC have some clinical-imaging similarities, and distinguishing them is conducive to reasonable diagnosis and treatment. Therefore, we will conduct clinical-imaging differentiation between CAP and pneumonia-type PC in the future to improve the integrity and practicality of the study. Because of poor operability, few patients in this study underwent cryptococcal culture to determine the Cryptococcus subtype. Fortunately, the treatments of Cryptococcus neoformans and Cryptococcus gattii are similar [30]. Small sample retrospective analysis cannot avoid selection bias and unstable statistical power. Large-scale longitudinal studies should be continued with refined immune assessment.

## Conclusion

We determined the clinical-imaging diversity of PC through clinical-imaging comparison, independent factor selection, diagnostic value validation, and therapeutic assessment. This closed-loop study showed that respiratory symptoms, inflammatory responses, bilateral distribution, and malignant CT signs were different between nodule-mass PC and pneumonia-type PC. Combining these clinical-imaging parameters could reveal the diversity of PC and improve diagnostic performance. Different CT morphological findings represented different infectious degrees and therapeutic strategies. Reasonable evaluation and intervention of PC can be achieved through CT morphology.

## Data Availability

Data of the current study is available from the corresponding author upon reasonable request.

## Abbreviations

HIV: Human immunodeficiency virus
PC: Pulmonary cryptococcosis
CT: Computer tomography
CrAg: Cryptococcal capsular antigen
OR: Odds ratio
95% CI: 95% Confidence interval
ICC: Inter-class correlation coefficient
ROC: Receiver operating characteristic
AUC: Area under curve
